# Supplementary material for: Characterizing the oligogenic architecture of plant growth phenotypes informs genomic selection approaches in a common wheat population
Source: BMC Genomics. 2021 May 31;22:402. doi: 10.1186/s12864-021-07574-6 (PMC8166015; doi:10.1186/s12864-021-07574-6)
Supplement: Supplementary file 1 — Additional file 1 Word document containing QTL results for spike length in Figure S1, KASP marker nucleotide sequences in Tables S1, S2 and S3 comparing prediction accuracies of the base regression model and the modified epistatic model for each phenotype. [file 12864_2021_7574_MOESM1_ESM.docx]

**Additional File 1**


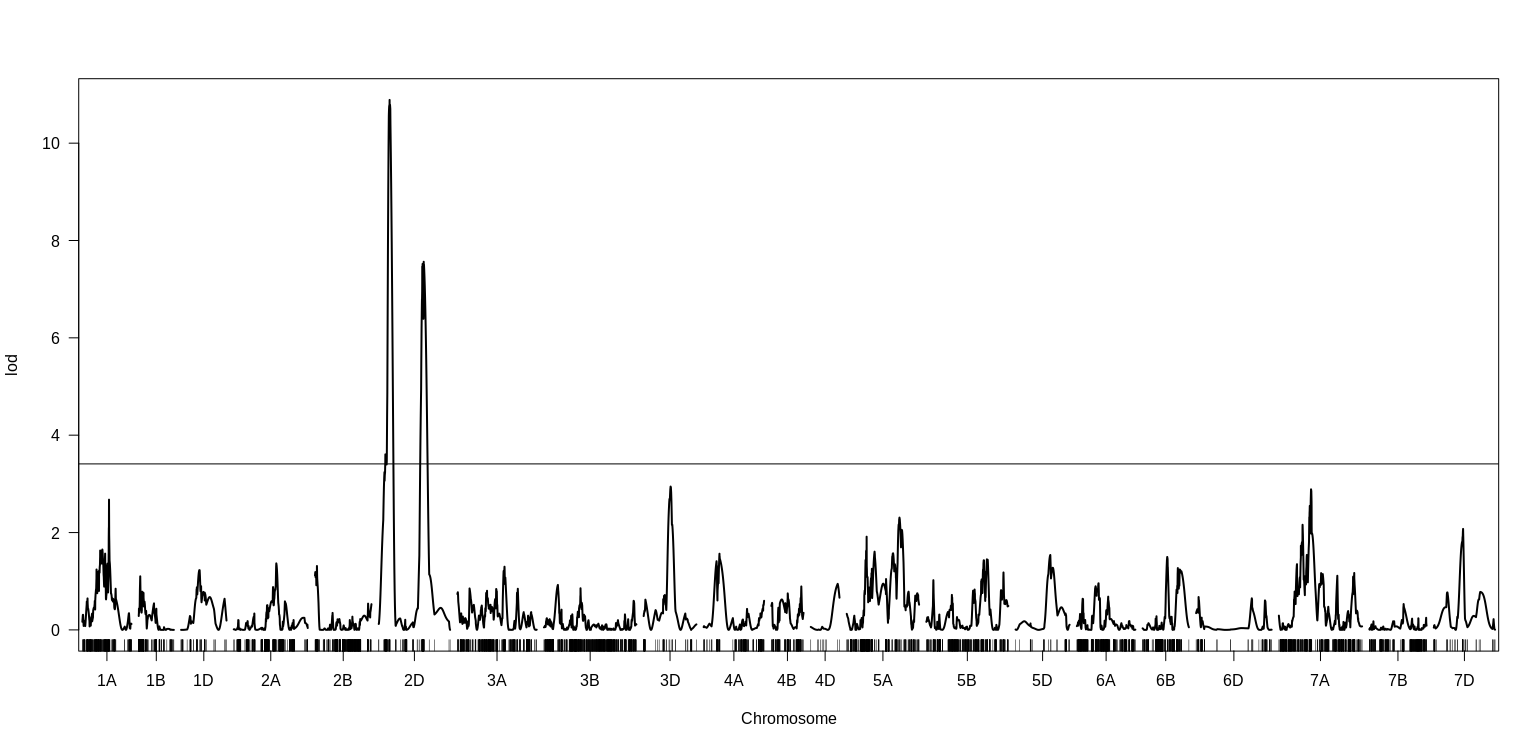


**Figure S1.** CIM QTL results for spike compactness in Raleigh 2019. Spike compactness was rated visually on a 1-5 scale, with 5 being most compact with a club wheat-like phenotype. Significance declared at a LOD of 3.41 for alpha = .05 based on a thousand permutations. The heading date locus on the short arm of chromosome 2D colocates with the major QTL, suggesting that it is likely *Rht8*.

| Marker_name | Chr | Sequence |
| --- | --- | --- |
| Rht-D1_A1 | 4D | GAAGGTGACCAAGTTCATGCTCATGGCCATCTCGAGCTRCTC |
| Rht-B1_A2 | 4D | GAAGGTCGGAGTCAACGGATTCATGGCCATCTCGAGCTRCTA |
| Rht-D1_C | 4D | CGGGTACAAGGTGCGCGCC |
| Ppd-D1_A1 | 2D | GAAGGTCGGAGTCAACGGATTAAGAGGAAACATGTTGGGGTCC |
| Ppd-D1_A2 | 2D | GAAGGTGACCAAGTTCATGCTCAAGGAAGTATGAGCAGCGGTT |
| Ppd-D1_C | 2D | GCCTCCCACTACACTGGGC |
| FTA2_A1 | 3A | GAAGGTGACCAAGTTCATGCTACGTCCACCGGCATCTTGGAC |
| FTA2_A2 | 3A | GAAGGTCGGAGTCAACGGATTCGTCCACCGGCATCTTGGAT |
| FTA2_C | 3A | GTACAGCTTCGGGGTACTGCTGTT |
| VrnA3_proDel_A1 | 7A | GAAGGTGACCAAGTTCATGCTCAGCTTACGCTTACTCTTGCTCCC |
| VrnA3_proDel_A2 | 7A | GAAGGTCGGAGTCAACGGATTCAGCTTACGCTTACTCTTGCTCCA |
| VrnA3_proDel_C | 7A | CTCCCGGCCATTTCCCCTTCC |
| B1_A1 | 5A | GAAGGTGACCAAGTTCATGCTAGCTACGGGCCCACTTRGACA |
| B1_A2 | 5A | GAAGGTCGGAGTCAACGGATTCTACGGGCCCACTTRGACG |
| B1_C1 | 5A | CCTGCGGGGCTCCCAGCAA |
| WAPOA1_Hap13vs2_A1 | 7A | GAAGGTGACCAAGTTCATGCTCTGATTATGGGCGGTTGATCTGC |
| WAPOA1_Hap13vs2_A2 | 7A | GAAGGTCGGAGTCAACGGATTTCTGATTATGGGCGGTTGATCTGT |
| WAPOA1_Hap13vs2_C1 | 7A | GACCAGCGCCGGCGACT |

**Table S1.** KASP marker sequences for variants that are tightly linked to or underly mapped QTL in this study.

| **Model** | **Ral18** | | **Kin19** | | **Pla19** | |
| --- | --- | --- | --- | --- | --- | --- |
|  | ***µ*** | ***sd*** | ***µ*** | ***sd*** | ***µ*** | ***sd*** |
| QTL Regression | 0.667 | 0.0175 | 0.636 | 0.0132 | 0.597 | 0.00521 |
| QTL Regres. + Interactions | 0.667 | 0.0176 | 0.636 | 0.0133 | 0.596 | 0.00531 |

**Table S2.** Prediction accuracies for heading date in the normal QTL regression model and a QTL regression model with the addition of detected epistatic interactions between QTL. Mean prediction accuracies and their standard deviations estimated in the replications of five-fold cross validations. The epistatic model did not outperform the standard regression model.

| **Model** | **Ral18** | | **Kin18** | | **Kin19** | |
| --- | --- | --- | --- | --- | --- | --- |
|  | ***µ*** | ***sd*** | ***µ*** | ***sd*** | ***µ*** | ***sd*** |
| QTL Regression | 0.776 | 0.00342 | 0.686 | 0.00351 | 0.785 | 0.00563 |
| QTL Regres. + Interactions | 0.775 | 0.00331 | 0.353 | 0.00352 | 0.785 | 0.00563 |

**Table S3.** Prediction accuracies for plant height in the normal QTL regression model and a QTL regression model with the addition of detected epistatic interactions between QTL. Mean prediction accuracies and their standard deviations estimated in the replications of five-fold cross validations. The epistatic model did not outperform the standard regression model.
